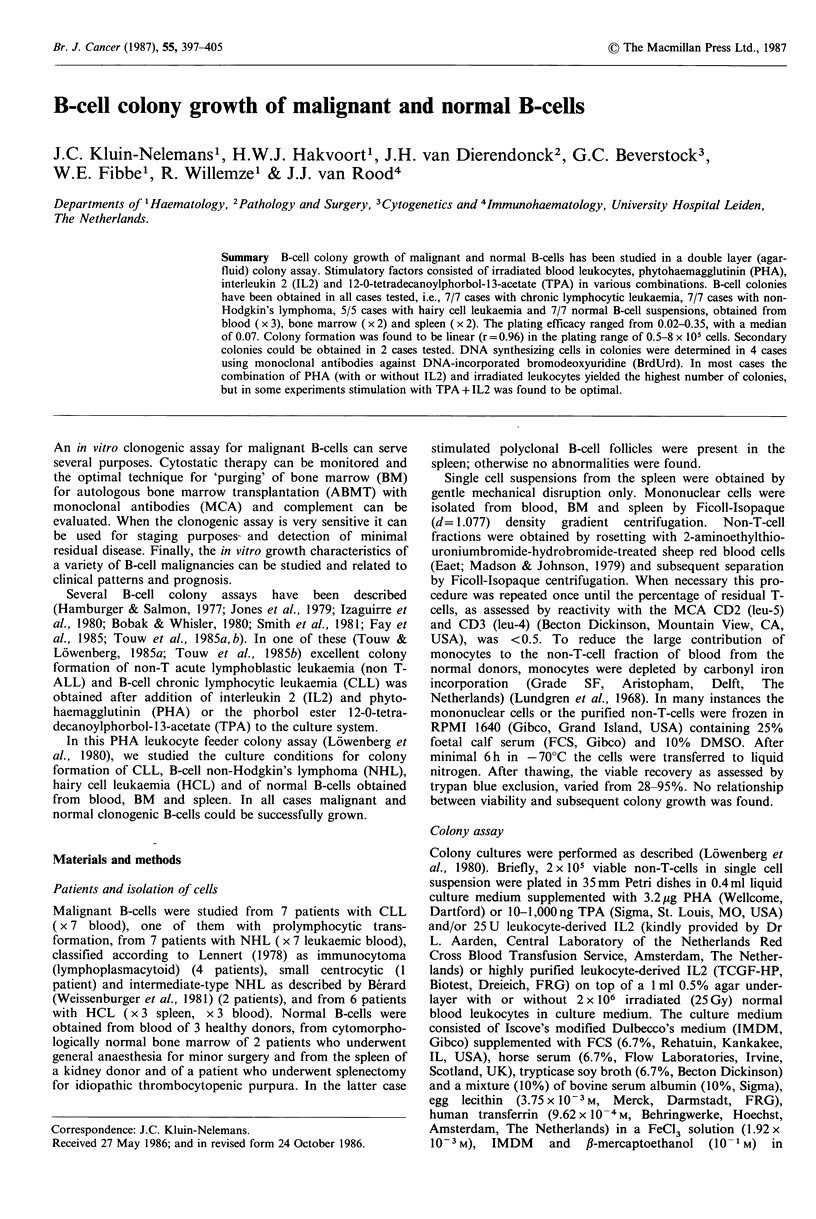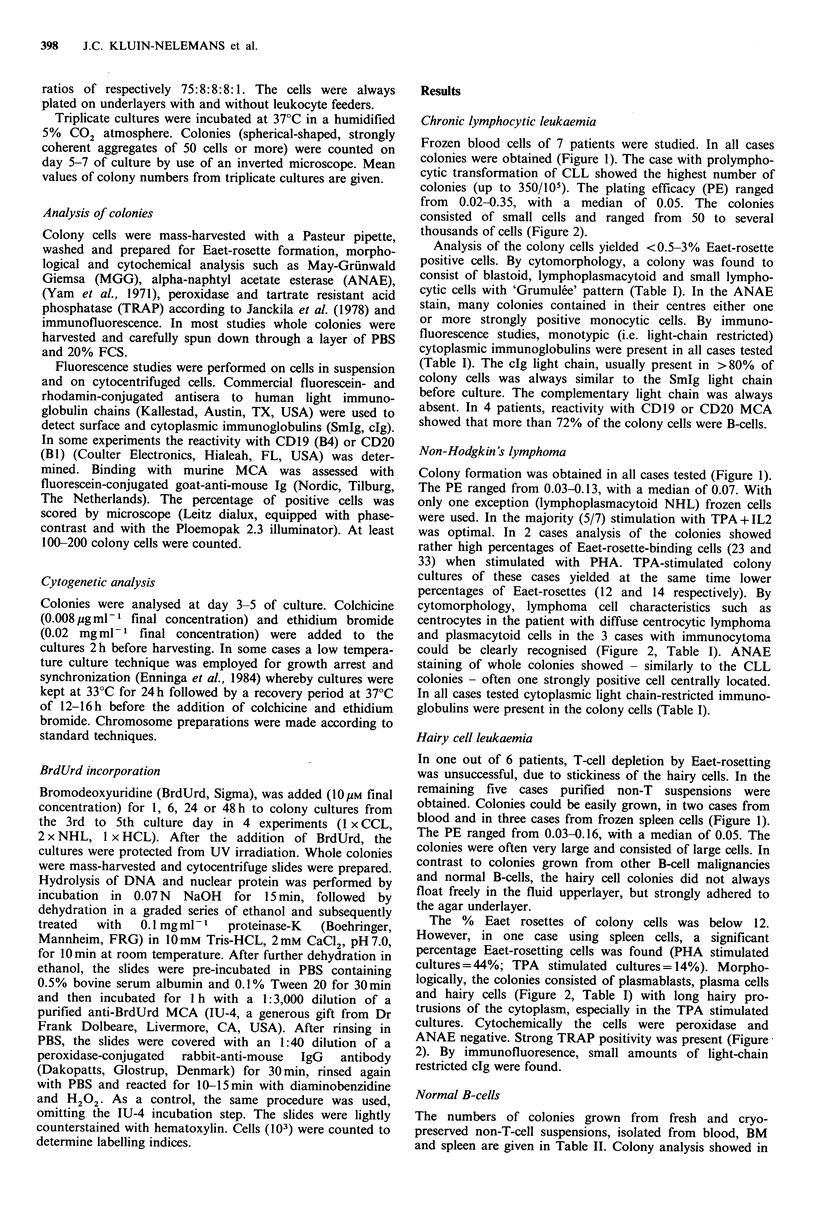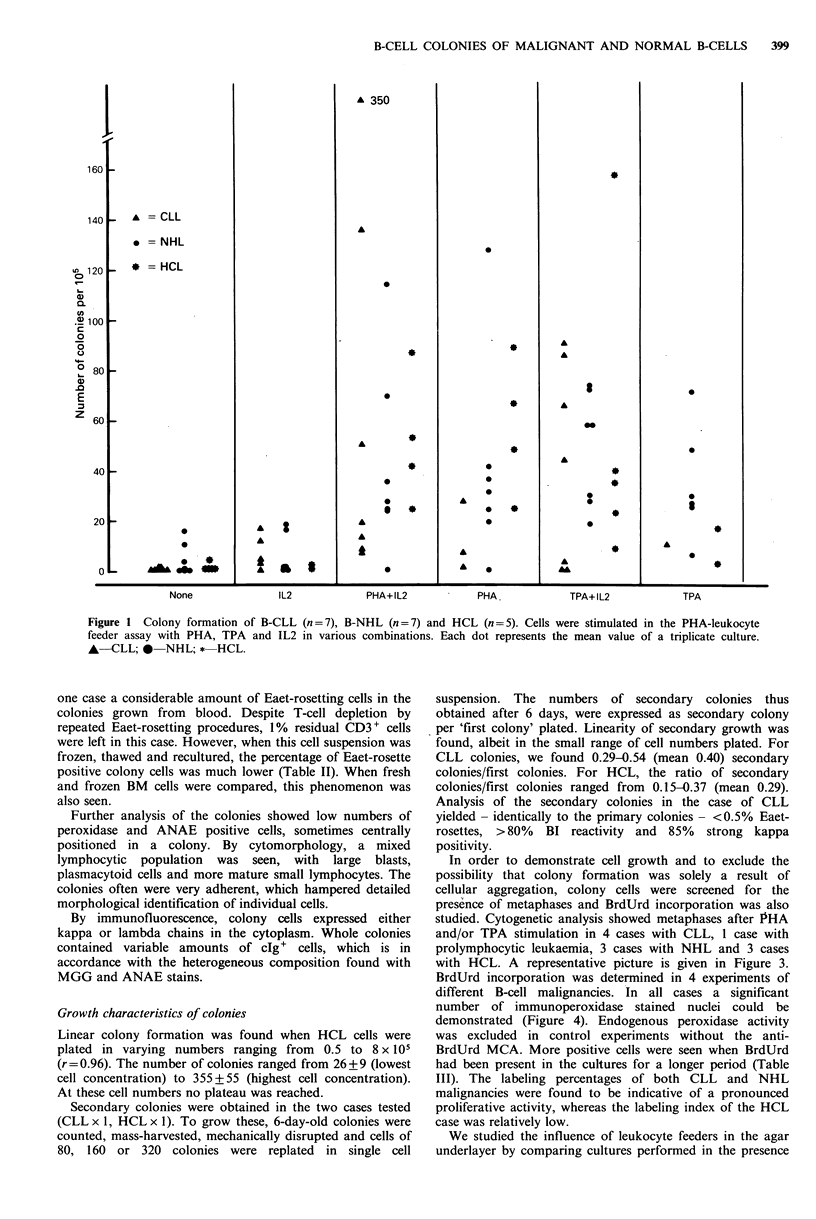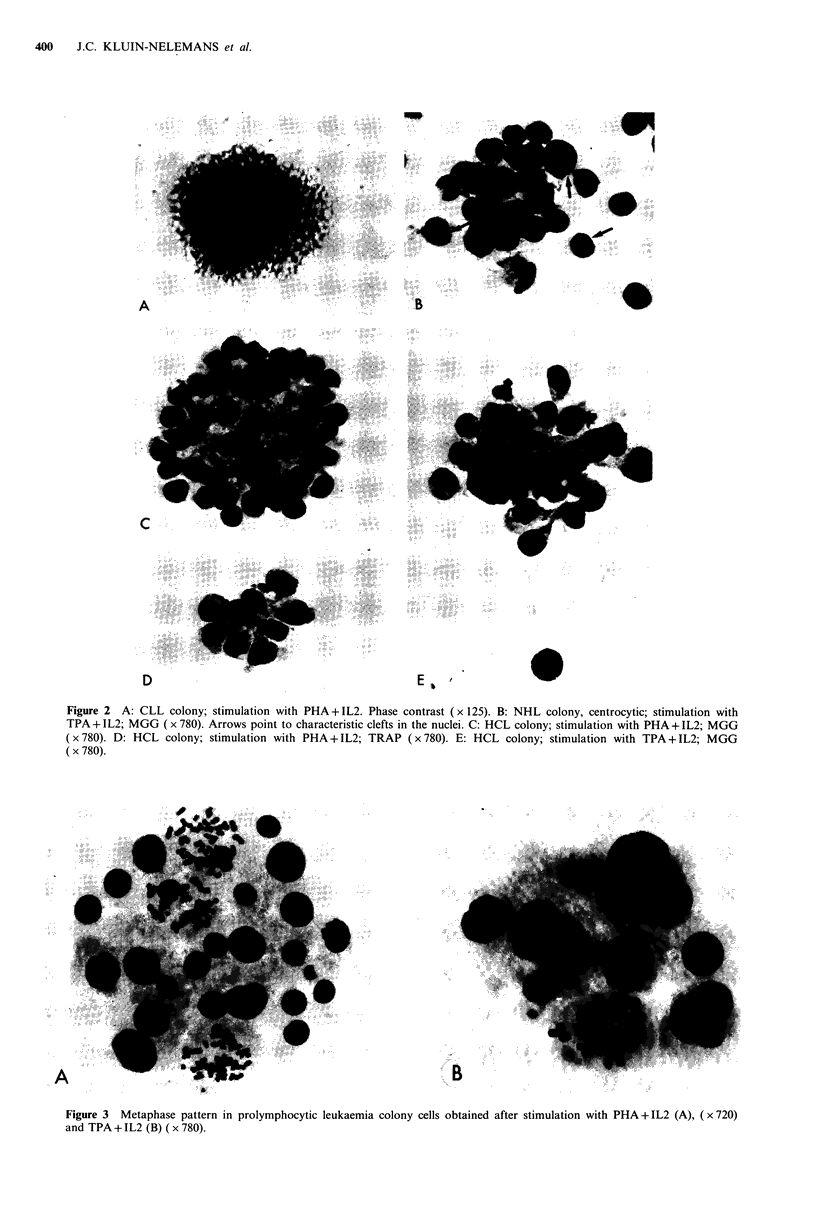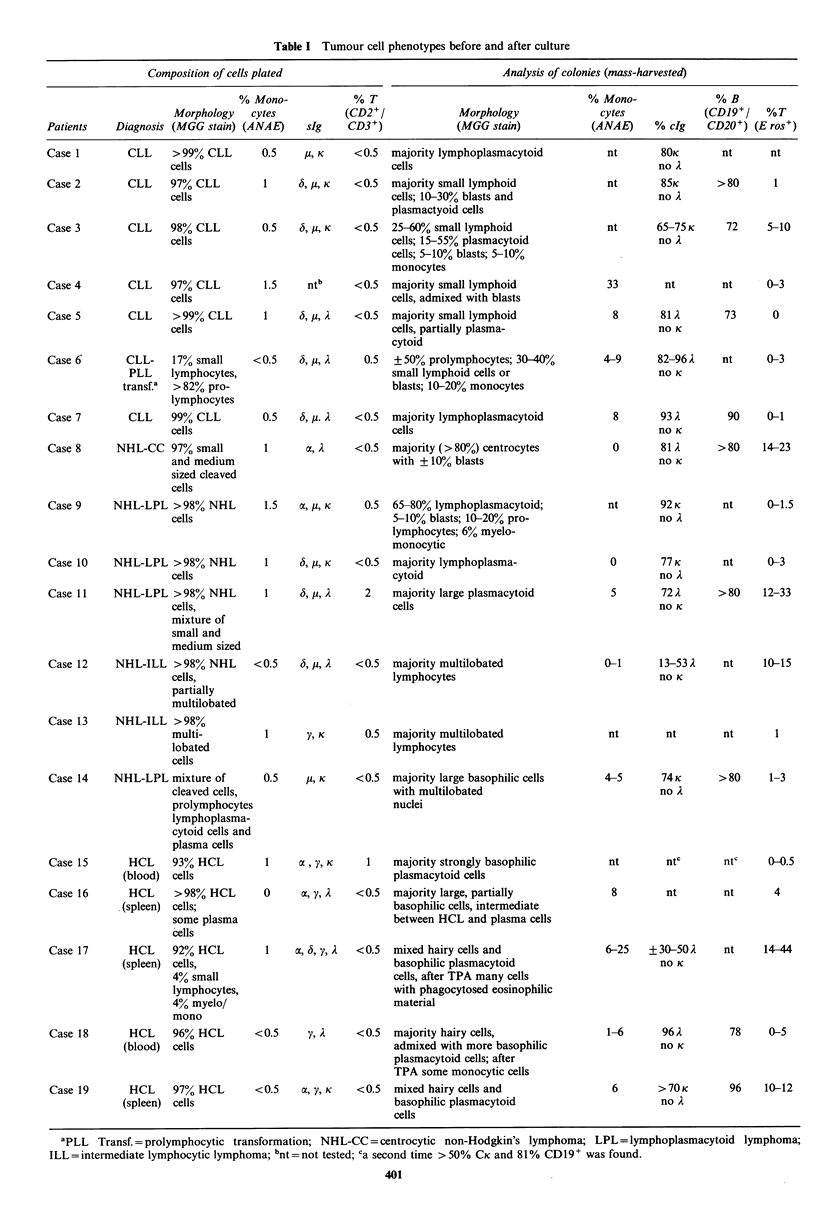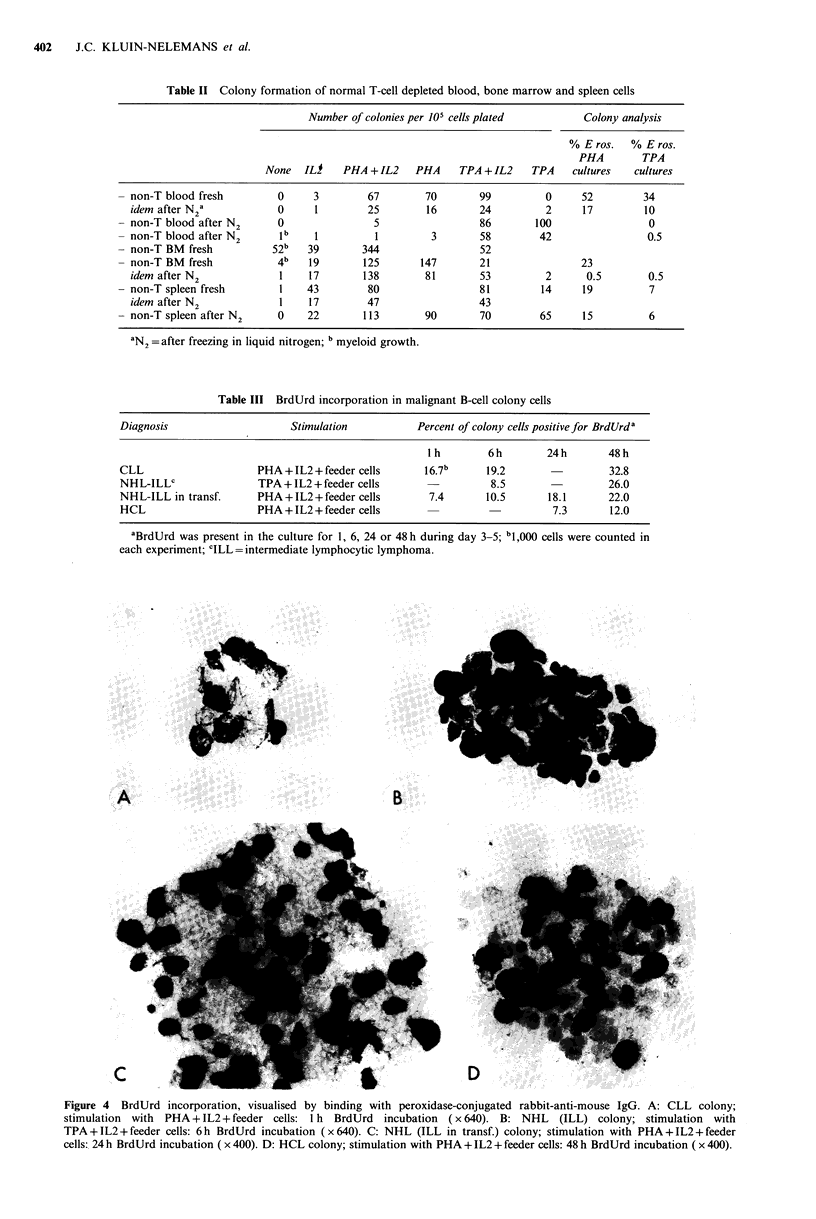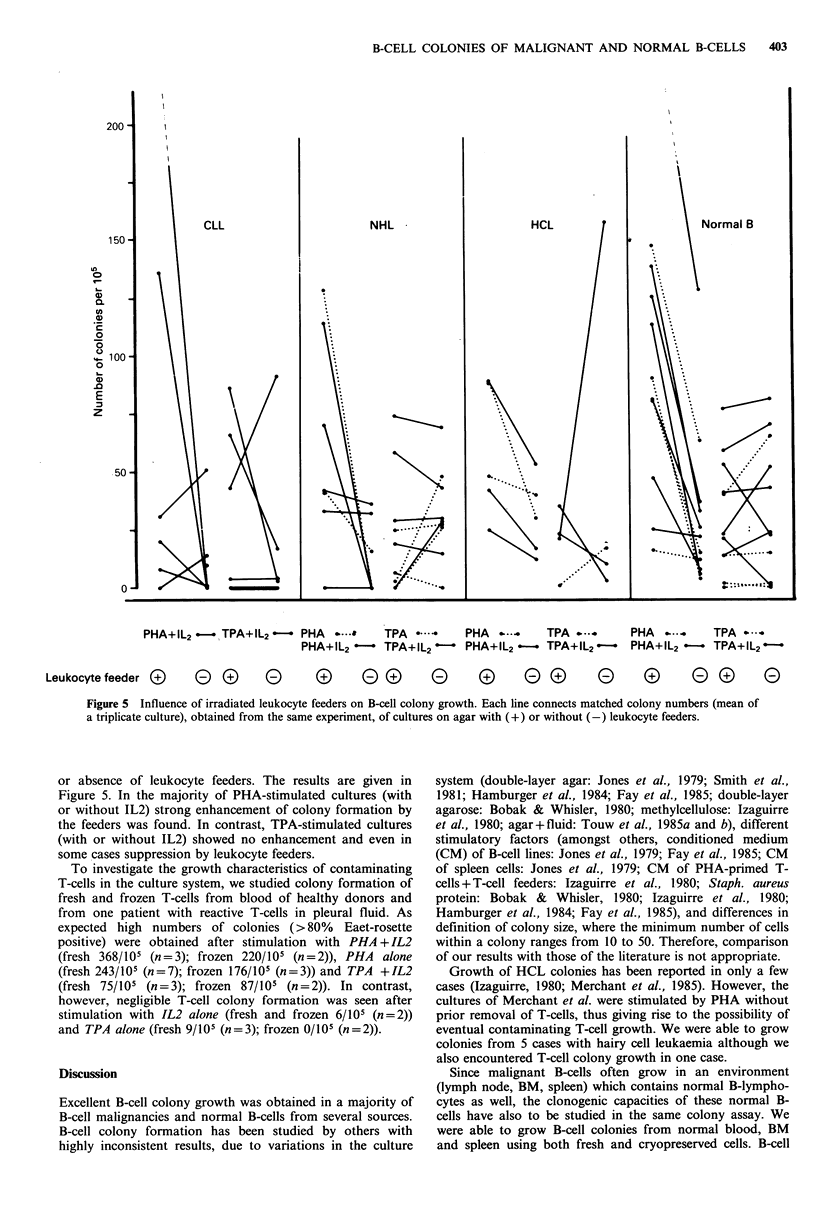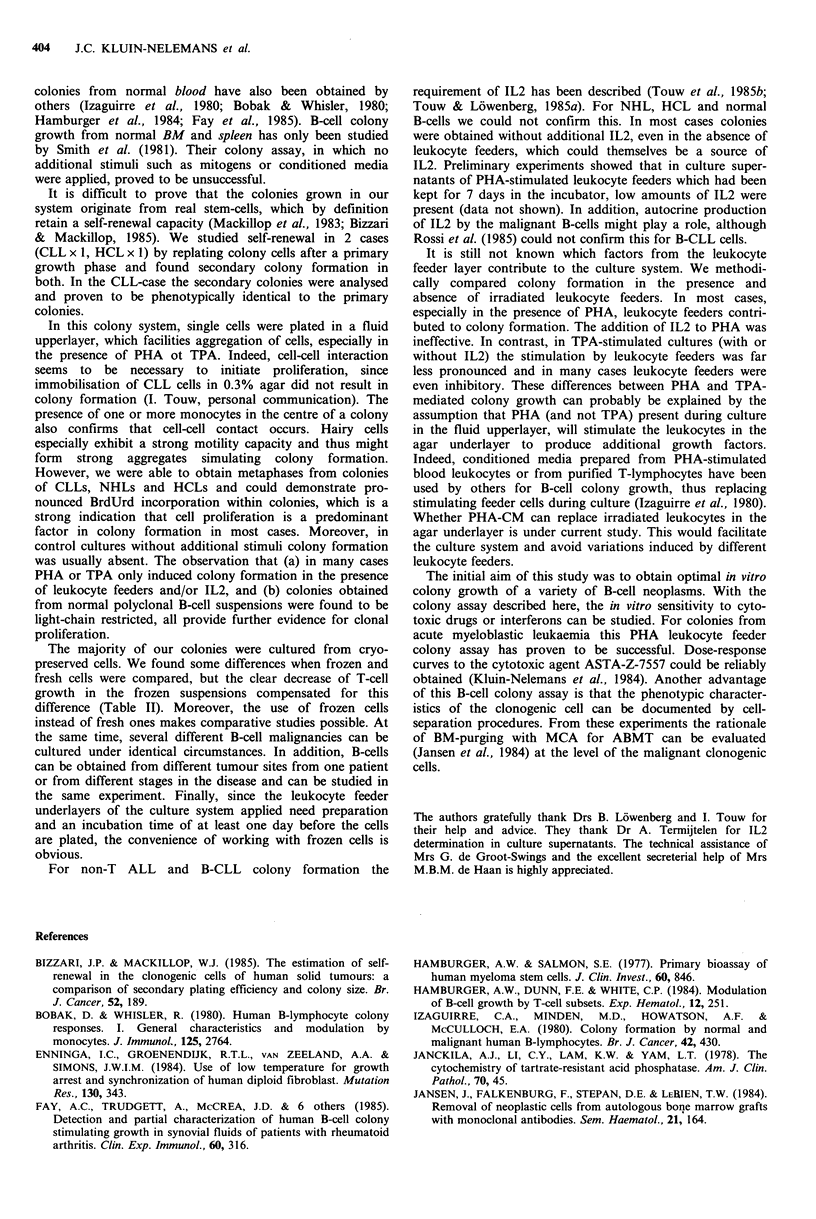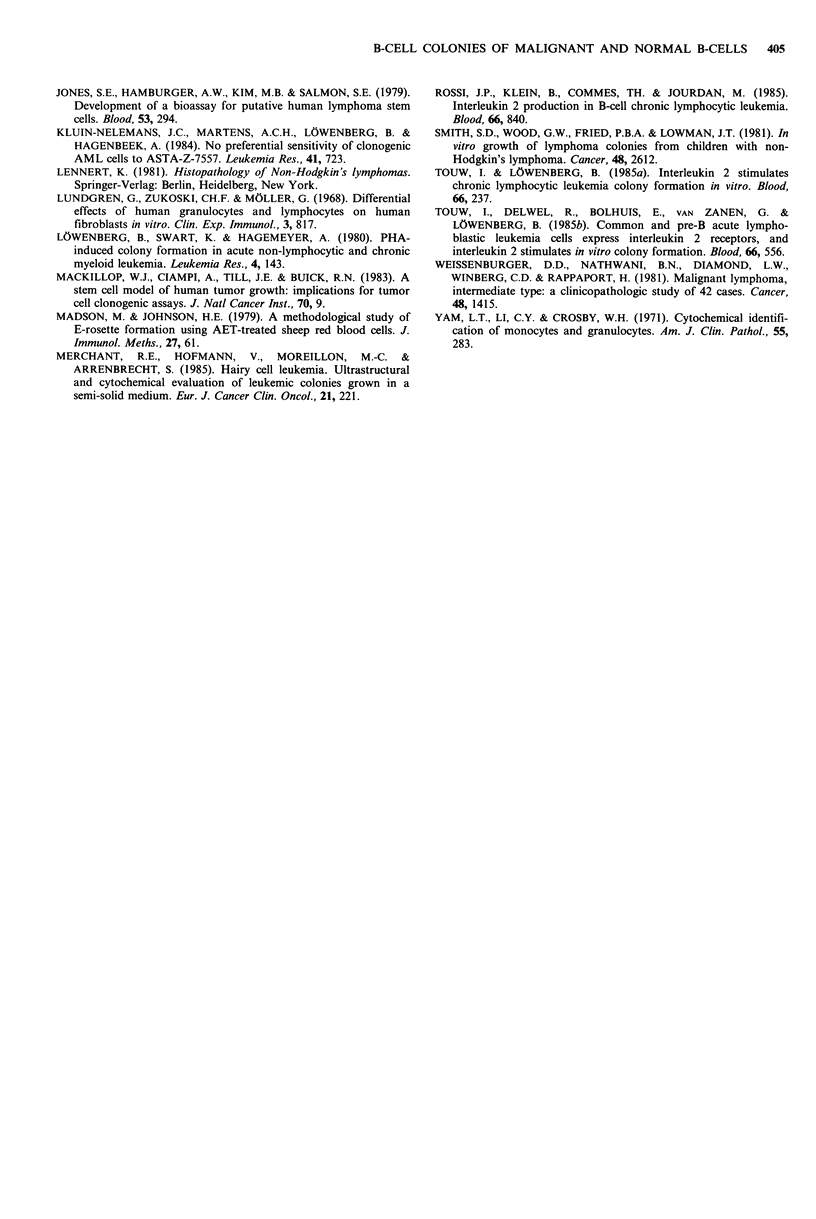# B-cell colony growth of malignant and normal B-cells.

**DOI:** 10.1038/bjc.1987.79

**Published:** 1987-04

**Authors:** J. C. Kluin-Nelemans, H. W. Hakvoort, J. H. van Dierendonck, G. C. Beverstock, W. E. Fibbe, R. Willemze, J. J. van Rood

## Abstract

**Images:**


 © The Macmillan Press Ltd., 1987

### B-cell colony growth of malignant and normal B-cells

J.C. Kluin-Nelemans[1], H.W.J. Hakvoort[1], J.H. van Dierendonck[2], G.C. Beverstock[3], W.E. Fibbe[1], R. Willemze[1] & J.J. van Rood[4]

*Departments of [1]Haematology, [2]Pathology and Surgery, [3]Cytogenetics and [4]Immunohaematology, University Hospital Leiden, The Netherlands.*

**Summary** B-cell colony growth of malignant and normal B-cells has been studied in a double layer (agar-fluid) colony assay. Stimulatory factors consisted of irradiated blood leukocytes, phytohaemagglutinin (PHA), interleukin 2 (IL2) and 12-0-tetradecanoylphorbol-13-acetate (TPA) in various combinations. B-cell colonies have been obtained in all cases tested, i.e., 7/7 cases with chronic lymphocytic leukaemia, 7/7 cases with non-Hodgkin's lymphoma, 5/5 cases with hairy cell leukaemia and 7/7 normal B-cell suspensions, obtained from blood ($\times 3$), bone marrow ($\times 2$) and spleen ($\times 2$). The plating efficacy ranged from 0.02–0.35, with a median of 0.07. Colony formation was found to be linear ($r=0.96$) in the plating range of $0.5–8 \times 10^5$ cells. Secondary colonies could be obtained in 2 cases tested. DNA synthesizing cells in colonies were determined in 4 cases using monoclonal antibodies against DNA-incorporated bromodeoxyuridine (BrdUrd). In most cases the combination of PHA (with or without IL2) and irradiated leukocytes yielded the highest number of colonies, but in some experiments stimulation with TPA+IL2 was found to be optimal.

An *in vitro* clonogenic assay for malignant B-cells can serve several purposes. Cytostatic therapy can be monitored and the optimal technique for 'purging' of bone marrow (BM) for autologous bone marrow transplantation (ABMT) with monoclonal antibodies (MCA) and complement can be evaluated. When the clonogenic assay is very sensitive it can be used for staging purposes and detection of minimal residual disease. Finally, the *in vitro* growth characteristics of a variety of B-cell malignancies can be studied and related to clinical patterns and prognosis.

Several B-cell colony assays have been described (Hamburger & Salmon, 1977; Jones *et al.*, 1979; Izaguirre *et al.*, 1980; Bobak & Whisler, 1980; Smith *et al.*, 1981; Fay *et al.*, 1985; Touw *et al.*, 1985*a,b*). In one of these (Touw & Löwenberg, 1985*a*; Touw *et al.*, 1985*b*) excellent colony formation of non-T acute lymphoblastic leukaemia (non T-ALL) and B-cell chronic lymphocytic leukaemia (CLL) was obtained after addition of interleukin 2 (IL2) and phytohaemagglutinin (PHA) or the phorbol ester 12-0-tetradecanoylphorbol-13-acetate (TPA) to the culture system.

In this PHA leukocyte feeder colony assay (Löwenberg *et al.*, 1980), we studied the culture conditions for colony formation of CLL, B-cell non-Hodgkin's lymphoma (NHL), hairy cell leukaemia (HCL) and of normal B-cells obtained from blood, BM and spleen. In all cases malignant and normal clonogenic B-cells could be successfully grown.

### Materials and methods

### *Patients and isolation of cells*

Malignant B-cells were studied from 7 patients with CLL ($\times 7$ blood), one of them with prolymphocytic transformation, from 7 patients with NHL ($\times 7$ leukaemic blood), classified according to Lennert (1978) as immunocytoma (lymphoplasmacytoid) (4 patients), small centrocytic (1 patient) and intermediate-type NHL as described by Bérard (Weissenburger *et al.*, 1981) (2 patients), and from 6 patients with HCL ($\times 3$ spleen, $\times 3$ blood). Normal B-cells were obtained from blood of 3 healthy donors, from cytomorphologically normal bone marrow of 2 patients who underwent general anaesthesia for minor surgery and from the spleen of a kidney donor and of a patient who underwent splenectomy for idiopathic thrombocytopenic purpura. In the latter case stimulated polyclonal B-cell follicles were present in the spleen; otherwise no abnormalities were found.

Single cell suspensions from the spleen were obtained by gentle mechanical disruption only. Mononuclear cells were isolated from blood, BM and spleen by Ficoll-Isopaque ($d=1.077$) density gradient centrifugation. Non-T-cell fractions were obtained by rosetting with 2-aminoethylthiouroniumbromide-hydrobromide-treated sheep red blood cells (Eaet; Madson & Johnson, 1979) and subsequent separation by Ficoll-Isopaque centrifugation. When necessary this procedure was repeated once until the percentage of residual T-cells, as assessed by reactivity with the MCA CD2 (leu-5) and CD3 (leu-4) (Becton Dickinson, Mountain View, CA, USA), was <0.5. To reduce the large contribution of monocytes to the non-T-cell fraction of blood from the normal donors, monocytes were depleted by carbonyl iron incorporation (Grade SF, Aristopham, Delft, The Netherlands) (Lundgren *et al.*, 1968). In many instances the mononuclear cells or the purified non-T-cells were frozen in RPMI 1640 (Gibco, Grand Island, USA) containing 25% foetal calf serum (FCS, Gibco) and 10% DMSO. After minimal 6 h in −70°C the cells were transferred to liquid nitrogen. After thawing, the viable recovery as assessed by trypan blue exclusion, varied from 28–95%. No relationship between viability and subsequent colony growth was found.

### *Colony assay*

Colony cultures were performed as described (Löwenberg *et al.*, 1980). Briefly, $2 \times 10^5$ viable non-T-cells in single cell suspension were plated in 35 mm Petri dishes in 0.4 ml liquid culture medium supplemented with 3.2 $\mu$g PHA (Wellcome, Dartford) or 10–1,000 ng TPA (Sigma, St. Louis, MO, USA) and/or 25 U leukocyte-derived IL2 (kindly provided by Dr L. Aarden, Central Laboratory of the Netherlands Red Cross Blood Transfusion Service, Amsterdam, The Netherlands) or highly purified leukocyte-derived IL2 (TCGF-HP, Biotest, Dreieich, FRG) on top of a 1 ml 0.5% agar underlayer with or without $2 \times 10^6$ irradiated (25 Gy) normal blood leukocytes in culture medium. The culture medium consisted of Iscove's modified Dulbecco's medium (IMDM, Gibco) supplemented with FCS (6.7%, Rehatuin, Kankakee, IL, USA), horse serum (6.7%, Flow Laboratories, Irvine, Scotland, UK), trypticase soy broth (6.7%, Becton Dickinson) and a mixture (10%) of bovine serum albumin (10%, Sigma), egg lecithin ($3.75 \times 10^{-3}$ M, Merck, Darmstadt, FRG), human transferrin ($9.62 \times 10^{-4}$ M, Behringwerke, Hoechst, Amsterdam, The Netherlands) in a $FeCl_3$ solution ($1.92 \times 10^{-3}$ M), IMDM and $\beta$-mercaptoethanol ($10^{-1}$ M) in

Correspondence: J.C. Kluin-Nelemans.
Received 27 May 1986; and in revised form 24 October 1986.

ratios of respectively 75:8:8:8:1. The cells were always plated on underlayers with and without leukocyte feeders.

Triplicate cultures were incubated at 37°C in a humidified 5% $CO_2$ atmosphere. Colonies (spherical-shaped, strongly coherent aggregates of 50 cells or more) were counted on day 5–7 of culture by use of an inverted microscope. Mean values of colony numbers from triplicate cultures are given.

*Analysis of colonies*

Colony cells were mass-harvested with a Pasteur pipette, washed and prepared for Eaet-rosette formation, morphological and cytochemical analysis such as May-Grünwald Giemsa (MGG), alpha-naphtyl acetate esterase (ANAE), (Yam *et al.*, 1971), peroxidase and tartrate resistant acid phosphatase (TRAP) according to Janckila *et al.* (1978) and immunofluorescence. In most studies whole colonies were harvested and carefully spun down through a layer of PBS and 20% FCS.

Fluorescence studies were performed on cells in suspension and on cytocentrifuged cells. Commercial fluorescein- and rhodamin-conjugated antisera to human light immunoglobulin chains (Kallestad, Austin, TX, USA) were used to detect surface and cytoplasmic immunoglobulins (SmIg, cIg). In some experiments the reactivity with CD19 (B4) or CD20 (B1) (Coulter Electronics, Hialeah, FL, USA) was determined. Binding with murine MCA was assessed with fluorescein-conjugated goat-anti-mouse Ig (Nordic, Tilburg, The Netherlands). The percentage of positive cells was scored by microscope (Leitz dialux, equipped with phase-contrast and with the Ploemopak 2.3 illuminator). At least 100–200 colony cells were counted.

*Cytogenetic analysis*

Colonies were analysed at day 3–5 of culture. Colchicine ($0.008\,\mu g\,ml^{-1}$ final concentration) and ethidium bromide ($0.02\ mg\,ml^{-1}$ final concentration) were added to the cultures 2 h before harvesting. In some cases a low temperature culture technique was employed for growth arrest and synchronization (Enninga *et al.*, 1984) whereby cultures were kept at 33°C for 24 h followed by a recovery period at 37°C of 12–16 h before the addition of colchicine and ethidium bromide. Chromosome preparations were made according to standard techniques.

*BrdUrd incorporation*

Bromodeoxyuridine (BrdUrd, Sigma), was added (10 $\mu$M final concentration) for 1, 6, 24 or 48 h to colony cultures from the 3rd to 5th culture day in 4 experiments (1 × CCL, 2 × NHL, 1 × HCL). After the addition of BrdUrd, the cultures were protected from UV irradiation. Whole colonies were mass-harvested and cytocentrifuge slides were prepared. Hydrolysis of DNA and nuclear protein was performed by incubation in 0.07 N NaOH for 15 min, followed by dehydration in a graded series of ethanol and subsequently treated with $0.1\,mg\,ml^{-1}$ proteinase-K (Boehringer, Mannheim, FRG) in 10 mM Tris-HCL, 2 mM $CaCl_2$, pH 7.0, for 10 min at room temperature. After further dehydration in ethanol, the slides were pre-incubated in PBS containing 0.5% bovine serum albumin and 0.1% Tween 20 for 30 min and then incubated for 1 h with a 1:3,000 dilution of a purified anti-BrdUrd MCA (IU-4, a generous gift from Dr Frank Dolbeare, Livermore, CA, USA). After rinsing in PBS, the slides were covered with an 1:40 dilution of a peroxidase-conjugated rabbit-anti-mouse IgG antibody (Dakopatts, Glostrup, Denmark) for 30 min, rinsed again with PBS and reacted for 10–15 min with diaminobenzidine and $H_2O_2$. As a control, the same procedure was used, omitting the IU-4 incubation step. The slides were lightly counterstained with hematoxylin. Cells ($10^3$) were counted to determine labelling indices.

### Results

*Chronic lymphocytic leukaemia*

Frozen blood cells of 7 patients were studied. In all cases colonies were obtained (Figure 1). The case with prolymphocytic transformation of CLL showed the highest number of colonies (up to $350/10^5$). The plating efficacy (PE) ranged from 0.02–0.35, with a median of 0.05. The colonies consisted of small cells and ranged from 50 to several thousands of cells (Figure 2).

Analysis of the colony cells yielded <0.5–3% Eaet-rosette positive cells. By cytomorphology, a colony was found to consist of blastoid, lymphoplasmacytoid and small lymphocytic cells with 'Grumulée' pattern (Table I). In the ANAE stain, many colonies contained in their centres either one or more strongly positive monocytic cells. By immunofluorescence studies, monotypic (i.e. light-chain restricted) cytoplasmic immunoglobulins were present in all cases tested (Table I). The cIg light chain, usually present in >80% of colony cells was always similar to the SmIg light chain before culture. The complementary light chain was always absent. In 4 patients, reactivity with CD19 or CD20 MCA showed that more than 72% of the colony cells were B-cells.

*Non-Hodgkin's lymphoma*

Colony formation was obtained in all cases tested (Figure 1). The PE ranged from 0.03–0.13, with a median of 0.07. With only one exception (lymphoplasmacytoid NHL) frozen cells were used. In the majority (5/7) stimulation with TPA + IL2 was optimal. In 2 cases analysis of the colonies showed rather high percentages of Eaet-rosette-binding cells (23 and 33) when stimulated with PHA. TPA-stimulated colony cultures of these cases yielded at the same time lower percentages of Eaet-rosettes (12 and 14 respectively). By cytomorphology, lymphoma cell characteristics such as centrocytes in the patient with diffuse centrocytic lymphoma and plasmacytoid cells in the 3 cases with immunocytoma could be clearly recognised (Figure 2, Table I). ANAE staining of whole colonies showed – similarly to the CLL colonies – often one strongly positive cell centrally located. In all cases tested cytoplasmic light chain-restricted immunoglobulins were present in the colony cells (Table I).

*Hairy cell leukaemia*

In one out of 6 patients, T-cell depletion by Eaet-rosetting was unsuccessful, due to stickiness of the hairy cells. In the remaining five cases purified non-T suspensions were obtained. Colonies could be easily grown, in two cases from blood and in three cases from frozen spleen cells (Figure 1). The PE ranged from 0.03–0.16, with a median of 0.05. The colonies were often very large and consisted of large cells. In contrast to colonies grown from other B-cell malignancies and normal B-cells, the hairy cell colonies did not always float freely in the fluid upperlayer, but strongly adhered to the agar underlayer.

The % Eaet rosettes of colony cells was below 12. However, in one case using spleen cells, a significant percentage Eaet-rosetting cells was found (PHA stimulated cultures = 44%; TPA stimulated cultures = 14%). Morphologically, the colonies consisted of plasmablasts, plasma cells and hairy cells (Figure 2, Table I) with long hairy protrusions of the cytoplasm, especially in the TPA stimulated cultures. Cytochemically the cells were peroxidase and ANAE negative. Strong TRAP positivity was present (Figure 2). By immunofluoresence, small amounts of light-chain restricted cIg were found.

*Normal B-cells*

The numbers of colonies grown from fresh and cryopreserved non-T-cell suspensions, isolated from blood, BM and spleen are given in Table II. Colony analysis showed in

**Figure 1** Colony formation of B-CLL ($n=7$), B-NHL ($n=7$) and HCL ($n=5$). Cells were stimulated in the PHA-leukocyte feeder assay with PHA, TPA and IL2 in various combinations. Each dot represents the mean value of a triplicate culture. ▲—CLL; ●—NHL; ∗—HCL.

one case a considerable amount of Eaet-rosetting cells in the colonies grown from blood. Despite T-cell depletion by repeated Eaet-rosetting procedures, 1% residual $CD3^+$ cells were left in this case. However, when this cell suspension was frozen, thawed and recultured, the percentage of Eaet-rosette positive colony cells was much lower (Table II). When fresh and frozen BM cells were compared, this phenomenon was also seen.

Further analysis of the colonies showed low numbers of peroxidase and ANAE positive cells, sometimes centrally positioned in a colony. By cytomorphology, a mixed lymphocytic population was seen, with large blasts, plasmacytoid cells and more mature small lymphocytes. The colonies often were very adherent, which hampered detailed morphological identification of individual cells.

By immunofluorescence, colony cells expressed either kappa or lambda chains in the cytoplasm. Whole colonies contained variable amounts of $cIg^+$ cells, which is in accordance with the heterogeneous composition found with MGG and ANAE stains.

### *Growth characteristics of colonies*

Linear colony formation was found when HCL cells were plated in varying numbers ranging from 0.5 to $8 \times 10^5$ ($r=0.96$). The number of colonies ranged from $26 \pm 9$ (lowest cell concentration) to $355 \pm 55$ (highest cell concentration). At these cell numbers no plateau was reached.

Secondary colonies were obtained in the two cases tested (CLL x 1, HCL x 1). To grow these, 6-day-old colonies were counted, mass-harvested, mechanically disrupted and cells of 80, 160 or 320 colonies were replated in single cell suspension. The numbers of secondary colonies thus obtained after 6 days, were expressed as secondary colony per 'first colony' plated. Linearity of secondary growth was found, albeit in the small range of cell numbers plated. For CLL colonies, we found 0.29–0.54 (mean 0.40) secondary colonies/first colonies. For HCL, the ratio of secondary colonies/first colonies ranged from 0.15–0.37 (mean 0.29). Analysis of the secondary colonies in the case of CLL yielded – identically to the primary colonies – $<0.5\%$ Eaet-rosettes, $>80\%$ BI reactivity and 85% strong kappa positivity.

In order to demonstrate cell growth and to exclude the possibility that colony formation was solely a result of cellular aggregation, colony cells were screened for the presence of metaphases and BrdUrd incorporation was also studied. Cytogenetic analysis showed metaphases after PHA and/or TPA stimulation in 4 cases with CLL, 1 case with prolymphocytic leukaemia, 3 cases with NHL and 3 cases with HCL. A representative picture is given in Figure 3. BrdUrd incorporation was determined in 4 experiments of different B-cell malignancies. In all cases a significant number of immunoperoxidase stained nuclei could be demonstrated (Figure 4). Endogenous peroxidase activity was excluded in control experiments without the anti-BrdUrd MCA. More positive cells were seen when BrdUrd had been present in the cultures for a longer period (Table III). The labeling percentages of both CLL and NHL malignancies were found to be indicative of a pronounced proliferative activity, whereas the labeling index of the HCL case was relatively low.

We studied the influence of leukocyte feeders in the agar underlayer by comparing cultures performed in the presence

**Figure 2** A: CLL colony; stimulation with PHA+IL2. Phase contrast (×125). B: NHL colony, centrocytic; stimulation with TPA+IL2; MGG (×780). Arrows point to characteristic clefts in the nuclei. C: HCL colony; stimulation with PHA+IL2; MGG (×780). D: HCL colony; stimulation with PHA+IL2; TRAP (×780). E: HCL colony; stimulation with TPA+IL2; MGG (×780).

**Figure 3** Metaphase pattern in prolymphocytic leukaemia colony cells obtained after stimulation with PHA+IL2 (A), (×720) and TPA+IL2 (B) (×780).

**Table I** Tumour cell phenotypes before and after culture

| | *Composition of cells plated* | | | | | *Analysis of colonies (mass-harvested)* | | | | |
|---|---|---|---|---|---|---|---|---|---|---|
| *Patients* | *Diagnosis* | *Morphology (MGG stain)* | *% Monocytes (ANAE)* | *sIg* | *% T ($CD2^+$/ $CD3^+$)* | *Morphology (MGG stain)* | *% Monocytes (ANAE)* | *% cIg* | *% B ($CD19^+$/ $CD20^+$)* | *%T (E $ros^+$)* |
| Case 1 | CLL | >99% CLL cells | 0.5 | μ, κ | <0.5 | majority lymphoplasmacytoid cells | nt | 80κ no λ | nt | nt |
| Case 2 | CLL | 97% CLL cells | 1 | δ, μ, κ | <0.5 | majority small lymphoid cells; 10–30% blasts and plasmactyoid cells | nt | 85κ no λ | >80 | 1 |
| Case 3 | CLL | 98% CLL cells | 0.5 | δ, μ, κ | <0.5 | 25–60% small lymphoid cells; 15–55% plasmacytoid cells; 5–10% blasts; 5–10% monocytes | nt | 65–75 κ no λ | 72 | 5–10 |
| Case 4 | CLL | 97% CLL cells | 1.5 | nt[b] | <0.5 | majority small lymphoid cells, admixed with blasts | 33 | nt | nt | 0–3 |
| Case 5 | CLL | >99% CLL cells | 1 | δ, μ, λ | <0.5 | majority small lymphoid cells, partially plasmacytoid | 8 | 81 λ no κ | 73 | 0 |
| Case 6 | CLL-PLL transf.[a] | 17% small lymphocytes, >82% prolymphocytes | <0.5 | δ, μ, λ | 0.5 | ±50% prolymphocytes; 30–40% small lymphoid cells or blasts; 10–20% monocytes | 4–9 | 82–96 λ no κ | nt | 0–3 |
| Case 7 | CLL | 99% CLL cells | 0.5 | δ, μ. λ | <0.5 | majority lymphoplasmacytoid cells | 8 | 93 λ no κ | 90 | 0–1 |
| Case 8 | NHL-CC | 97% small and medium sized cleaved cells | 1 | α, λ | <0.5 | majority (>80%) centrocytes with ±10% blasts | 0 | 81 λ no κ | >80 | 14–23 |
| Case 9 | NHL-LPL | >98% NHL cells | 1.5 | α, μ, κ | 0.5 | 65–80% lymphoplasmacytoid; 5–10% blasts; 10–20% prolymphocytes; 6% myelomonocytic | nt | 92 κ no λ | nt | 0–1.5 |
| Case 10 | NHL-LPL | >98% NHL cells | 1 | δ, μ, κ | <0.5 | majority lymphoplasmacytoid | 0 | 77 κ no λ | nt | 0–3 |
| Case 11 | NHL-LPL | >98% NHL cells, mixture of small and medium sized | 1 | δ, μ, λ | 2 | majority large plasmacytoid cells | 5 | 72 λ no κ | >80 | 12–33 |
| Case 12 | NHL-ILL | >98% NHL cells, partially multilobated | <0.5 | δ, μ, λ | <0.5 | majority multilobated lymphocytes | 0–1 | 13–53 λ no κ | nt | 10–15 |
| Case 13 | NHL-ILL | >98% multilobated cells | 1 | γ, κ | 0.5 | majority multilobated lymphocytes | nt | nt | nt | 1 |
| Case 14 | NHL-LPL | mixture of cleaved cells, prolymphocytes lymphoplasmacytoid cells and plasma cells | 0.5 | μ, κ | <0.5 | majority large basophilic cells with multilobated nuclei | 4–5 | 74 κ no λ | >80 | 1–3 |
| Case 15 | HCL (blood) | 93% HCL cells | 1 | α, γ, κ | 1 | majority strongly basophilic plasmacytoid cells | nt | nt[c] | nt[c] | 0–0.5 |
| Case 16 | HCL (spleen) | >98% HCL cells; some plasma cells | 0 | α, γ, λ | <0.5 | majority large, partially basophilic cells, intermediate between HCL and plasma cells | 8 | nt | nt | 4 |
| Case 17 | HCL (spleen) | 92% HCL cells, 4% small lymphocytes, 4% myelo/mono | 1 | α, δ, γ, λ | <0.5 | mixed hairy cells and basophilic plasmacytoid cells, after TPA many cells with phagocytosed eosinophilic material | 6–25 | ±30–50 λ no κ | nt | 14–44 |
| Case 18 | HCL (blood) | 96% HCL cells | <0.5 | γ, λ | <0.5 | majority hairy cells, admixed with more basophilic plasmacytoid cells; after TPA some monocytic cells | 1–6 | 96 λ no κ | 78 | 0–5 |
| Case 19 | HCL (spleen) | 97% HCL cells | <0.5 | α, γ, κ | <0.5 | mixed hairy cells and basophilic plasmacytoid cells | 6 | >70 κ no λ | 96 | 10–12 |

[a]PLL Transf. = prolymphocytic transformation; NHL-CC = centrocytic non-Hodgkin's lymphoma; LPL = lymphoplasmacytoid lymphoma; ILL = intermediate lymphocytic lymphoma; [b]nt = not tested; [c]a second time >50% Cκ and 81% $CD19^+$ was found.

**Table II** Colony formation of normal T-cell depleted blood, bone marrow and spleen cells

| | *Number of colonies per $10^5$ cells plated* | | | | | | *Colony analysis* | |
|---|---|---|---|---|---|---|---|---|
| | *None* | *IL2* | *PHA+IL2* | *PHA* | *TPA+IL2* | *TPA* | *% E ros. PHA cultures* | *% E ros. TPA cultures* |
| – non-T blood fresh | 0 | 3 | 67 | 70 | 99 | 0 | 52 | 34 |
| *idem* after $N_2$[a] | 0 | 1 | 25 | 16 | 24 | 2 | 17 | 10 |
| – non-T blood after $N_2$ | 0 | | 5 | | 86 | 100 | | 0 |
| – non-T blood after $N_2$ | 1[b] | 1 | 1 | 3 | 58 | 42 | | 0.5 |
| – non-T BM fresh | 52[b] | 39 | 344 | | 52 | | | |
| – non-T BM fresh | 4[b] | 19 | 125 | 147 | 21 | | 23 | |
| *idem* after $N_2$ | 1 | 17 | 138 | 81 | 53 | 2 | 0.5 | 0.5 |
| – non-T spleen fresh | 1 | 43 | 80 | | 81 | 14 | 19 | 7 |
| *idem* after $N_2$ | 1 | 17 | 47 | | 43 | | | |
| – non-T spleen after $N_2$ | 0 | 22 | 113 | 90 | 70 | 65 | 15 | 6 |

[a]$N_2$ = after freezing in liquid nitrogen; [b] myeloid growth.

**Table III** BrdUrd incorporation in malignant B-cell colony cells

| *Diagnosis* | *Stimulation* | *Percent of colony cells positive for BrdUrd*[a] | | | |
|---|---|---|---|---|---|
| | | 1 h | 6 h | 24 h | 48 h |
| CLL | PHA+IL2+feeder cells | 16.7[b] | 19.2 | — | 32.8 |
| NHL-ILL[c] | TPA+IL2+feeder cells | — | 8.5 | — | 26.0 |
| NHL-ILL in transf. | PHA+IL2+feeder cells | 7.4 | 10.5 | 18.1 | 22.0 |
| HCL | PHA+IL2+feeder cells | — | — | 7.3 | 12.0 |

[a]BrdUrd was present in the culture for 1, 6, 24 or 48 h during day 3–5; [b]1,000 cells were counted in each experiment; [c]ILL = intermediate lymphocytic lymphoma.

**Figure 4** BrdUrd incorporation, visualised by binding with peroxidase-conjugated rabbit-anti-mouse IgG. A: CLL colony; stimulation with PHA+IL2+feeder cells: 1 h BrdUrd incubation (×640). B: NHL (ILL) colony; stimulation with TPA+IL2+feeder cells: 6 h BrdUrd incubation (×640). C: NHL (ILL in transf.) colony; stimulation with PHA+IL2+feeder cells: 24 h BrdUrd incubation (×400). D: HCL colony; stimulation with PHA+IL2+feeder cells: 48 h BrdUrd incubation (×400).

**Figure 5** Influence of irradiated leukocyte feeders on B-cell colony growth. Each line connects matched colony numbers (mean of a triplicate culture), obtained from the same experiment, of cultures on agar with (+) or without (−) leukocyte feeders.

or absence of leukocyte feeders. The results are given in Figure 5. In the majority of PHA-stimulated cultures (with or without IL2) strong enhancement of colony formation by the feeders was found. In contrast, TPA-stimulated cultures (with or without IL2) showed no enhancement and even in some cases suppression by leukocyte feeders.

To investigate the growth characteristics of contaminating T-cells in the culture system, we studied colony formation of fresh and frozen T-cells from blood of healthy donors and from one patient with reactive T-cells in pleural fluid. As expected high numbers of colonies (>80% Eaet-rosette positive) were obtained after stimulation with *PHA+IL2* (fresh $368/10^5$ ($n=3$); frozen $220/10^5$ ($n=2$)), *PHA alone* (fresh $243/10^5$ ($n=7$); frozen $176/10^5$ ($n=3$)) and *TPA +IL2* (fresh $75/10^5$ ($n=3$); frozen $87/10^5$ ($n=2$)). In contrast, however, negligible T-cell colony formation was seen after stimulation with *IL2 alone* (fresh and frozen $6/10^5$ ($n=2$)) and *TPA alone* (fresh $9/10^5$ ($n=3$); frozen $0/10^5$ ($n=2$)).

### Discussion

Excellent B-cell colony growth was obtained in a majority of B-cell malignancies and normal B-cells from several sources. B-cell colony formation has been studied by others with highly inconsistent results, due to variations in the culture system (double-layer agar: Jones *et al.*, 1979; Smith *et al.*, 1981; Hamburger *et al.*, 1984; Fay *et al.*, 1985; double-layer agarose: Bobak & Whisler, 1980; methylcellulose: Izaguirre *et al.*, 1980; agar+fluid: Touw *et al.*, 1985*a* and *b*), different stimulatory factors (amongst others, conditioned medium (CM) of B-cell lines: Jones *et al.*, 1979; Fay *et al.*, 1985; CM of spleen cells: Jones *et al.*, 1979; CM of PHA-primed T-cells+T-cell feeders: Izaguirre *et al.*, 1980; *Staph. aureus* protein: Bobak & Whisler, 1980; Izaguirre *et al.*, 1980; Hamburger *et al.*, 1984; Fay *et al.*, 1985), and differences in definition of colony size, where the minimum number of cells within a colony ranges from 10 to 50. Therefore, comparison of our results with those of the literature is not appropriate.

Growth of HCL colonies has been reported in only a few cases (Izaguirre, 1980; Merchant *et al.*, 1985). However, the cultures of Merchant *et al.* were stimulated by PHA without prior removal of T-cells, thus giving rise to the possibility of eventual contaminating T-cell growth. We were able to grow colonies from 5 cases with hairy cell leukaemia although we also encountered T-cell colony growth in one case.

Since malignant B-cells often grow in an environment (lymph node, BM, spleen) which contains normal B-lymphocytes as well, the clonogenic capacities of these normal B-cells have also to be studied in the same colony assay. We were able to grow B-cell colonies from normal blood, BM and spleen using both fresh and cryopreserved cells. B-cell colonies from normal *blood* have also been obtained by others (Izaguirre *et al.*, 1980; Bobak & Whisler, 1980; Hamburger *et al.*, 1984; Fay *et al.*, 1985). B-cell colony growth from normal *BM* and *spleen* has only been studied by Smith *et al.* (1981). Their colony assay, in which no additional stimuli such as mitogens or conditioned media were applied, proved to be unsuccessful.

It is difficult to prove that the colonies grown in our system originate from real stem-cells, which by definition retain a self-renewal capacity (Mackillop *et al.*, 1983; Bizzari & Mackillop, 1985). We studied self-renewal in 2 cases (CLL x 1, HCL x 1) by replating colony cells after a primary growth phase and found secondary colony formation in both. In the CLL-case the secondary colonies were analysed and proven to be phenotypically identical to the primary colonies.

In this colony system, single cells were plated in a fluid upperlayer, which facilities aggregation of cells, especially in the presence of PHA ot TPA. Indeed, cell-cell interaction seems to be necessary to initiate proliferation, since immobilisation of CLL cells in 0.3% agar did not result in colony formation (I. Touw, personal communication). The presence of one or more monocytes in the centre of a colony also confirms that cell-cell contact occurs. Hairy cells especially exhibit a strong motility capacity and thus might form strong aggregates simulating colony formation. However, we were able to obtain metaphases from colonies of CLLs, NHLs and HCLs and could demonstrate pronounced BrdUrd incorporation within colonies, which is a strong indication that cell proliferation is a predominant factor in colony formation in most cases. Moreover, in control cultures without additional stimuli colony formation was usually absent. The observation that (a) in many cases PHA or TPA only induced colony formation in the presence of leukocyte feeders and/or IL2, and (b) colonies obtained from normal polyclonal B-cell suspensions were found to be light-chain restricted, all provide further evidence for clonal proliferation.

The majority of our colonies were cultured from cryopreserved cells. We found some differences when frozen and fresh cells were compared, but the clear decrease of T-cell growth in the frozen suspensions compensated for this difference (Table II). Moreover, the use of frozen cells instead of fresh ones makes comparative studies possible. At the same time, several different B-cell malignancies can be cultured under identical circumstances. In addition, B-cells can be obtained from different tumour sites from one patient or from different stages in the disease and can be studied in the same experiment. Finally, since the leukocyte feeder underlayers of the culture system applied need preparation and an incubation time of at least one day before the cells are plated, the convenience of working with frozen cells is obvious.

For non-T ALL and B-CLL colony formation the requirement of IL2 has been described (Touw *et al.*, 1985*b*; Touw & Löwenberg, 1985*a*). For NHL, HCL and normal B-cells we could not confirm this. In most cases colonies were obtained without additional IL2, even in the absence of leukocyte feeders, which could themselves be a source of IL2. Preliminary experiments showed that in culture supernatants of PHA-stimulated leukocyte feeders which had been kept for 7 days in the incubator, low amounts of IL2 were present (data not shown). In addition, autocrine production of IL2 by the malignant B-cells might play a role, although Rossi *et al.* (1985) could not confirm this for B-CLL cells.

It is still not known which factors from the leukocyte feeder layer contribute to the culture system. We methodically compared colony formation in the presence and absence of irradiated leukocyte feeders. In most cases, especially in the presence of PHA, leukocyte feeders contributed to colony formation. The addition of IL2 to PHA was ineffective. In contrast, in TPA-stimulated cultures (with or without IL2) the stimulation by leukocyte feeders was far less pronounced and in many cases leukocyte feeders were even inhibitory. These differences between PHA and TPA-mediated colony growth can probably be explained by the assumption that PHA (and not TPA) present during culture in the fluid upperlayer, will stimulate the leukocytes in the agar underlayer to produce additional growth factors. Indeed, conditioned media prepared from PHA-stimulated blood leukocytes or from purified T-lymphocytes have been used by others for B-cell colony growth, thus replacing stimulating feeder cells during culture (Izaguirre *et al.*, 1980). Whether PHA-CM can replace irradiated leukocytes in the agar underlayer is under current study. This would facilitate the culture system and avoid variations induced by different leukocyte feeders.

The initial aim of this study was to obtain optimal *in vitro* colony growth of a variety of B-cell neoplasms. With the colony assay described here, the *in vitro* sensitivity to cytotoxic drugs or interferons can be studied. For colonies from acute myeloblastic leukaemia this PHA leukocyte feeder colony assay has proven to be successful. Dose-response curves to the cytotoxic agent ASTA-Z-7557 could be reliably obtained (Kluin-Nelemans *et al.*, 1984). Another advantage of this B-cell colony assay is that the phenotypic characteristics of the clonogenic cell can be documented by cell-separation procedures. From these experiments the rationale of BM-purging with MCA for ABMT can be evaluated (Jansen *et al.*, 1984) at the level of the malignant clonogenic cells.

The authors gratefully thank Drs B. Löwenberg and I. Touw for their help and advice. They thank Dr A. Termijtelen for IL2 determination in culture supernatants. The technical assistance of Mrs G. de Groot-Swings and the excellent secreterial help of Mrs M.B.M. de Haan is highly appreciated.

### References

BIZZARI, J.P. & MACKILLOP, W.J. (1985). The estimation of self-renewal in the clonogenic cells of human solid tumours: a comparison of secondary plating efficiency and colony size. *Br. J. Cancer*, **52**, 189.

BOBAK, D. & WHISLER, R. (1980). Human B-lymphocyte colony responses. I. General characteristics and modulation by monocytes. *J. Immunol.*, **125**, 2764.

ENNINGA, I.C., GROENENDIJK, R.T.L., VAN ZEELAND, A.A. & SIMONS, J.W.I.M. (1984). Use of low temperature for growth arrest and synchronization of human diploid fibroblast. *Mutation Res.*, **130**, 343.

FAY, A.C., TRUDGETT, A., McCREA, J.D. & 6 others (1985). Detection and partial characterization of human B-cell colony stimulating growth in synovial fluids of patients with rheumatoid arthritis. *Clin. Exp. Immunol.*, **60**, 316.

HAMBURGER, A.W. & SALMON, S.E. (1977). Primary bioassay of human myeloma stem cells. *J. Clin. Invest.*, **60**, 846.

HAMBURGER, A.W., DUNN, F.E. & WHITE, C.P. (1984). Modulation of B-cell growth by T-cell subsets. *Exp. Hematol.*, **12**, 251.

IZAGUIRRE, C.A., MINDEN, M.D., HOWATSON, A.F. & McCULLOCH, E.A. (1980). Colony formation by normal and malignant human B-lymphocytes. *Br. J. Cancer*, **42**, 430.

JANCKILA, A.J., LI, C.Y., LAM, K.W. & YAM, L.T. (1978). The cytochemistry of tartrate-resistant acid phosphatase. *Am. J. Clin. Pathol.*, **70**, 45.

JANSEN, J., FALKENBURG, F., STEPAN, D.E. & LEBIEN, T.W. (1984). Removal of neoplastic cells from autologous bone marrow grafts with monoclonal antibodies. *Sem. Haematol.*, **21**, 164.

JONES, S.E., HAMBURGER, A.W., KIM, M.B. & SALMON, S.E. (1979). Development of a bioassay for putative human lymphoma stem cells. *Blood,* **53,** 294.

KLUIN-NELEMANS, J.C., MARTENS, A.C.H., LÖWENBERG, B. & HAGENBEEK, A. (1984). No preferential sensitivity of clonogenic AML cells to ASTA-Z-7557. *Leukemia Res.,* **41,** 723.

LENNERT, K. (1981). *Histopathology of Non-Hodgkin's lymphomas.* Springer-Verlag: Berlin, Heidelberg, New York.

LUNDGREN, G., ZUKOSKI, CH.F. & MÖLLER, G. (1968). Differential effects of human granulocytes and lymphocytes on human fibroblasts *in vitro. Clin. Exp. Immunol.,* **3,** 817.

LÖWENBERG, B., SWART, K. & HAGEMEYER, A. (1980). PHA-induced colony formation in acute non-lymphocytic and chronic myeloid leukemia. *Leukemia Res.,* **4,** 143.

MACKILLOP, W.J., CIAMPI, A., TILL, J.E. & BUICK, R.N. (1983). A stem cell model of human tumor growth: implications for tumor cell clonogenic assays. *J. Natl Cancer Inst.,* **70,** 9.

MADSON, M. & JOHNSON, H.E. (1979). A methodological study of E-rosette formation using AET-treated sheep red blood cells. *J. Immunol. Meths.,* **27,** 61.

MERCHANT, R.E., HOFMANN, V., MOREILLON, M.-C. & ARRENBRECHT, S. (1985). Hairy cell leukemia. Ultrastructural and cytochemical evaluation of leukemic colonies grown in a semi-solid medium. *Eur. J. Cancer Clin. Oncol.,* **21,** 221.

ROSSI, J.P., KLEIN, B., COMMES, TH. & JOURDAN, M. (1985). Interleukin 2 production in B-cell chronic lymphocytic leukemia. *Blood,* **66,** 840.

SMITH, S.D., WOOD, G.W., FRIED, P.B.A. & LOWMAN, J.T. (1981). *In vitro* growth of lymphoma colonies from children with non-Hodgkin's lymphoma. *Cancer,* **48,** 2612.

TOUW, I. & LÖWENBERG, B. (1985*a*). Interleukin 2 stimulates chronic lymphocytic leukemia colony formation *in vitro. Blood,* **66,** 237.

TOUW, I., DELWEL, R., BOLHUIS, E., VAN ZANEN, G. & LÖWENBERG, B. (1985*b*). Common and pre-B acute lymphoblastic leukemia cells express interleukin 2 receptors, and interleukin 2 stimulates *in vitro* colony formation. *Blood,* **66,** 556.

WEISSENBURGER, D.D., NATHWANI, B.N., DIAMOND, L.W., WINBERG, C.D. & RAPPAPORT, H. (1981). Malignant lymphoma, intermediate type: a clinicopathologic study of 42 cases. *Cancer,* **48,** 1415.

YAM, L.T., LI, C.Y. & CROSBY, W.H. (1971). Cytochemical identification of monocytes and granulocytes. *Am. J. Clin. Pathol.,* **55,** 283.